# Brain-Derived Extracellular Vesicle microRNA Signatures Associated with In Utero and Postnatal Oxycodone Exposure

**DOI:** 10.3390/cells9010021

**Published:** 2019-12-19

**Authors:** Farah Shahjin, Rahul S. Guda, Victoria L. Schaal, Katherine Odegaard, Alexander Clark, Austin Gowen, Peng Xiao, Steven J. Lisco, Gurudutt Pendyala, Sowmya V. Yelamanchili

**Affiliations:** 1 Department of Anesthesiology, University of Nebraska Medical Center, Omaha, NE 68198, USA; farah.shahjin@unmc.edu (F.S.); rahulguda@college.harvard.edu (R.S.G.); vicki.schaal@unmc.edu (V.L.S.); katherine.odegaard@unmc.edu (K.O.); alexclark@creighton.edu (A.C.); austin.gowen@unmc.edu (A.G.); steven.lisco@unmc.edu (S.J.L.); 2 Department of Genetics Cell Biology and Anatomy, University of Nebraska Medical Center, Omaha, NE 68198, USA; peng.xiao@unmc.edu

**Keywords:** oxycodone, in utero, postnatal, brain derived EVs, RNA-Seq

## Abstract

Oxycodone (oxy) is a semi-synthetic opioid commonly used as a pain medication that is also a widely abused prescription drug. While very limited studies have examined the effect of in utero oxy (IUO) exposure on neurodevelopment, a significant gap in knowledge is the effect of IUO compared with postnatal oxy (PNO) exposure on synaptogenesis—a key process in the formation of synapses during brain development—in the exposed offspring. One relatively unexplored form of cell–cell communication associated with brain development in response to IUO and PNO exposure are extracellular vesicles (EVs). EVs are membrane-bound vesicles that serve as carriers of cargo, such as microRNAs (miRNAs). Using RNA-Seq analysis, we identified distinct brain-derived extracellular vesicle (BDEs) miRNA signatures associated with IUO and PNO exposure, including their gene targets, regulating key functional pathways associated with brain development to be more impacted in the IUO offspring. Further treatment of primary 14-day in vitro (DIV) neurons with IUO BDEs caused a significant reduction in spine density compared to treatment with BDEs from PNO and saline groups. In summary, our studies identified for the first time, key BDE miRNA signatures in IUO- and PNO-exposed offspring, which could impact their brain development as well as synaptic function.

## 1. Introduction

The abuse of prescription opiates has significantly escalated over the last two decades in the United States [1,2]. Several thousand individuals are hospitalized every year due to the non-medical use of prescription opiates, posing a significant public health challenge [2]. The current study focuses on prescription opioid oxycodone (oxy). Oxy is generally prescribed to treat multiple types of pain and has an affinity to bind to both mu- and kappa-opioid receptors [3]. Given its ability to cross the blood–brain barrier, higher concentrations of oxy can easily be achieved in the brain [4,5], contributing both to its analgesic properties as well as its risk of dependency and subsequent addiction. Adding another layer of complexity is the increased risk of oxy dependency during pregnancy and postpartum, which are linked to maladaptive behaviors and cognitive deficits in exposed offspring. There is a lack of studies that have compared the effect of in utero oxy (IUO) and postnatal oxy (PNO) exposure on synaptogenesis—a key process of the formation of synapses during brain development—in the exposed offspring. Synapses are key communication points between neurons, which play a critical role in the regulation of neurotransmission and brain plasticity [6]. 

One relatively unexplored form of cell–cell communication associated with brain development in response to IUO and PNO exposure is extracellular vesicles (EVs) [7,8,9]. These membrane-bound vesicles, comprised of exosomes and microvesicles, originate from the multivesicular bodies and plasma membrane, respectively. Furthermore, these EVs, which carry a repertoire of cargo, including microRNAs (miRNAs), are shown to induce inflammation and subsequent neuronal damage, thus serving as key mediators of pathogenesis in several neurological and neurodegenerative disorders [10,11]. The main objective of this study was to identify differentially expressed BDEs miRNAs using RNA sequencing (RNA-Seq) and evaluate their impact on synaptic architecture during a key stage of brain development. 

## 2. Materials and Methods

### 2.1. Animals

Male and female Sprague Dawley rats were obtained from Charles River Laboratories Inc. (Wilmington, MA, USA) and group housed in a 12-h light–dark cycle and fed ad libitum. All procedures and protocols were approved by the Institutional Animal Care and Use Committee of the University of Nebraska Medical Center and conducted in accordance with the National Institutes of Health Guide for the Care and Use of Laboratory Animals.

### 2.2. Oxycodone Treatment

The development of the IUO treatment paradigm was adapted from a previously published study [12]. Nulliparous female (64–70 days of age) Sprague Dawley rats were treated with oxycodone HCl (Sigma Aldrich, St. Louis, MO, USA) dissolved in saline or saline vehicle via oral gavage. An ascending dosing procedure was used wherein doses of 10 mg/kg/day oxy were orally gavaged for 5 days followed by 0.5 mg/kg/day escalation for 10 days to a final dose of 15 mg/kg/day, after which females were mated with proven male breeders, and the treatment regimen continued throughout mating, gestation, and parturition until weaning. Because the half-life of oxy is relatively short and drug distribution can be altered by pregnancy, dams were monitored daily for signs of opiate withdrawal, such as weight loss, diarrhea, and irritability, throughout gestation. For the PNO paradigm, dams were orally gavaged with 15 mg/kg/day of oxy after parturition until weaning. For the current study, brains from saline, IUO, and PNO offspring were harvested at postnatal day 14 (P14) and stored at –80 °C until subsequent use. 

### 2.3. Brain-Derived Extracellular Vesicle Isolation

BDEs were isolated from P14 brains from the three treatment groups as described in our previous studies [13,14]. Briefly, brain tissue was dissected and digested in 20 units/mL papain in Hibernate A (Life Technologies), followed by immediate addition of cold Hibernate A to stop the enzymatic digestion. Cell, debris, and other larger particles were removed by multiple centrifugations (300× *g*, 2000× *g*, 10,000× *g*) and filtration through a 0.22-μm filter. EVs were further concentrated by ultracentrifugation (100,000× g) at 4 °C, followed by its purification using density gradient separation technique. For the density gradient separation, sucrose concentrations ranging from 0.25 to 2 M were used, where the concentrated EVs pellet was resuspended in the 0.95 M layer and centrifuged at 200,000× *g* for 16 h at 4 °C. Fractions enriched with EVs were subjected to further ultracentrifugation at 100,000× *g* to obtain the EVs pellet.

### 2.4. RNA Isolation and Sequencing

The EVs pellets were then subjected to miRNA extraction using the mirVana miRNA Isolation Kit (Life Technologies) following the manufacturer’s instructions. RNA samples were then sent to LC Sciences (Houston, TX, USA) for miRNA sequencing. 

### 2.5. Western Blot

The purity of the isolated BDEs was assessed using specific antibodies by western blot as described in our previous studies [14,15,16]. Briefly, exosome protein lysates were prepared using RIPA buffer with 1% SDS and protease-phosphatase inhibitor. Protein quantification was done using the Pierce BCA protein assay kit (Thermo Scientific) following which 40 μg of protein (for positive markers) and 10 μg (for negative marker) from three control P14 brain lysates or synaptosomes were run on 4% to 12% Bis-Tris gels (Invitrogen) under reducing (Hsp70, flotillin, calnexin, DRD1, and DRD2) and non-reducing conditions (CD81) followed by transfer using iBLOT2 (Invitrogen). Nonspecific antibody binding was done using Superblock^TM^ blocking buffer (Thermo Scientific). Immunoblotting was carried out overnight at 4 °C with primary antibodies against the positive BDE markers Hsp70 (1:1000, Sigma), flotillin-1 (1:1000, Abcam), CD81 (1:500, Bio-Rad), DRD1 (1:1000, Chemicon), DRD2 (1:1500, NovusBio), and the negative marker calnexin (1:1000, Abcam) followed by secondary antibody (1:2500 HRP conjugated anti-mouse IgG for Hsp70, 1:2500 HRP conjugated anti-rabbit IgG for flotillin-1, DRD1 and DRD2, 1:1500 HRP conjugated anti-hamster IgG for CD81, and 1:2000 HRP conjugated anti-rabbit IgG for calnexin). Blots were developed with 1:1 solution of Radiance Chemiluminescent Substrate and Luminol/Enhancer (Azure Biosystems) and visualized using c300 imaging system (Azure Biosystems). Images were quantified using the ImageJ software. 

### 2.6. Transmission Electron Microscopy (TEM)

TEM was performed as described in previous studies [14,17]. Briefly, BDEs pellet was resuspended in 1x PBS. Then, 10 µL of the BDEs suspension was mixed with 90 µL of TEM fix buffer (2% glutaraldehyde, 2% paraformaldehyde, and 0.1 M phosphate buffer). A 10-μL drop of BDEs-buffer solution was placed on 200-mesh copper grids coated with formvar and silicon monoxide and allowed to sit for 2 min. The excess solution was drawn off by filter paper, and the remaining thin film of sample was allowed to air dry for 2 min. A drop of NanoVan (Nanoprobes, New York, NY, USA) negative stain was placed on the grid for 1 min. The excess negative stain was then drawn off by filter paper and allowed to dry for at least 1 min before being imaged. Grids were examined on a Tecnai G2 TEM (built by FEI, Hillsboro, OR, USA) operated at 80 Kv. An Advanced Microscopy Techniques digital imaging system was used for image acquisition. 

### 2.7. Bioinformatics

Differentially expressed miRNAs were mapped to their targets using the ‘miRNA Target Filter’ in Ingenuity Pathway Analysis software (Qiagenbioinformatics.com). Only those targets that were either experimentally characterized or predicted by TargetScan [18] with high confidence were selected. Target genes associated with developmental disorders, neurological disorders, and psychological disorders were chosen for further functional characterization using Clue-GO plug-in module [19] in Cytoscape software [20]. The ‘biological process’ option in Clue-Go analysis was used to visualize the categories of target gene functions.

### 2.8. Primary Rat Neuronal Cultures and BDEs Treatment

Primary cortical neurons were cultured as described in our previous studies [16]. Briefly, rat cortical neurons from embryonic day 18 Sprague Dawley rat (Brain Bits LLC, Springfield, IL, USA) were plated at a density of 0.25 × 10^6^ cells per well onto a 24-well plate containing one poly-d-lysine coated coverslip per well. Cells were cultured in Neurobasal media containing 0.5 mM l-glutamine and B27 supplement (Life Technologies), at 37 °C in a humidified atmosphere of a 5% CO_2_ incubator with periodic half exchange of the medium every three days. After 14 days in vitro (DIV), cells were treated for 24 h with a 1:1000 ratio of cells to BDEs (1.5 × 10^8^ particles) (from the three different treatment groups), as measured by nanoparticle tracking analysis, followed by confocal imaging of dendritic spines. 

### 2.9. Immunostaining of Dendritic Spines

At 24 h post-BDEs treatment, cells were fixed in 4% paraformaldehyde and immunostained for spine analysis by confocal imaging. Briefly, neurons were permeabilized with 0.25% Tween-20 in 1x PBS for 15 min, followed by blocking in 0.5% normal goat serum, 0.25% Tween-20, and 1% bovine serum albumin in 1x PBS for 1 h at room temperature. All primary and secondary antibodies were diluted in 1% normal goat serum, 0.25% Tween-20, and 1% bovine serum albumin in 1x PBS. Neurons were incubated with primary antibodies MAP2 (Rabbit, 1:200, Thermo Scientific, Rockford, IL, USA) and Drebrin (Mouse 1:1000, MBL, Woburn, MA, USA) overnight at 4 °C. Coverslips were then washed three times with 1x PBS. Secondary antibodies (Alexa 488, Chicken-Anti-Rabbit, 1:500, and Alexa 568 Donkey-Anti-Mouse, 1:500, Thermo Scientific, Rockford, IL, USA) and DAPI were used to incubate the neurons for 1 h at room temperature. Cells were washed with 1x PBS and mounted with ProLong Gold antifade (Invitrogen P36930) on Superfrost Plus charged microscope slides (Fisherbrand). 

### 2.10. Confocal Imaging and Image J Analysis

Z-Stack images (pixel size 0.1 µm with a 0.1-µm Z Step) were acquired with a Zeiss Observer Z1 fluorescent microscope with ApoTome (63x oil objective, NA 1.4). Then, 10-stacked images were compressed together in ImageJ using the Z Projection function. All three-color channels were later merged and 50-µm lengths of dendrites were selected and manually counted in the Image J image viewer pane. Three images per coverslip were captured and five dendrites per image were used in spine counts and subsequent analysis. 

### 2.11. Crude Synaptosome Isolation 

At P14, one pup (either sex) was randomly selected from each litter of each group for brain dissection. The animal was anesthetized using isoflurane (Piramal Critical Care, Bethlehem, PA, USA) then cervically dislocated to ensure death. The prefrontal cortices were dissected from the brain and homogenized in 1 mL of lysis buffer (0.32 M sucrose, 5 mM HEPES, 0.1 mM EDTA, deionized water, and one protein/phosphatase inhibitor tablet) (Invitrogen, Halethorpe, MD, USA) (Roche Applied Science, Indianapolis, IN, USA). A slow spin was performed at 1.8 rpm for five minutes at 4 °C. The supernatant was removed, and the pellet was dissolved in 500 µL of lysis buffer. A second spin was performed at 12,000 rpm for 20 min at 4 °C. Supernatant was removed, and the pellet was resuspended in 500 µL of lysis buffer. The crude synaptosomal pellet was used for BCA and western blot experiments. 

### 2.12. Statistical Analyses

All data represented in the manuscript are reported as mean ± SEM. Student’s *t*-test was used to test the miRNA differential expression between the comparison groups (saline vs. PNO; saline vs. IUO; and PNO vs. IUO) with a significance criterion of *p* ≤ 0.05. Data were analyzed using the Graph Pad Prism (La Jolla, CA, USA) software.

### 2.13. Bioinformatics for 2D Hierarchical Clustering

2D hierarchical clustering heatmaps were performed by heatmap package in R. Statistical normalization data of log2 (normalized miRNA expression value +0.0001) were used for the clustering to avoid any nonsense log2(0) values.

## 3. Results

### 3.1. Purity and Characterization of Isolated BDEs

**Figure 1:**
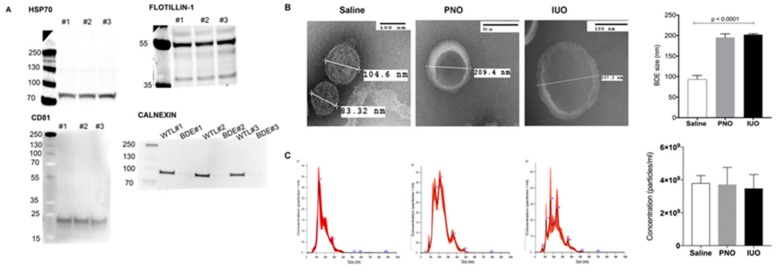
Characterization of BDEs. (**A**) Western blot analysis on BDEs from three P14 saline animals (#1, #2, and #3) show the expression of the positive markers CD81, flotillin-1, and Hsp70. Note the negative marker was enriched in the whole tissue lysate (WTL) but absent in the EVs fraction (BDE). (**B**) BDEs isolated using a sucrose density gradient from the three groups were characterized using TEM. (**C**) Nano tracking analysis (NTA) showing the different size distribution; no significant changes in concentrations of isolated BDEs from the three groups was observed.

To determine the purity of the BDEs, we isolated total EVs protein from three P14 saline animals and examined them against specific positive and negative markers via western blot analysis as described in the minimal information for studies of EVs outlined by the International Society for Extracellular Vesicles [21]. As seen in Figure 1A, the positive markers CD81, flotillin-1, and Hsp70 were detected in the EVs fraction while the negative marker calnexin was absent in the EVs fraction compared to the total lysates, thus lending credibility to our isolation technique. TEM [17] was used to characterize the BDEs that revealed a cup-like appearance of vesicles. While the saline BDEs were characterized at ~100 nm, a significant increase was observed in both the PNO and IUO groups (Figure 1B,C). However, no difference in the total number of EV particles was observed in the different groups. These data suggest that oxy exposure can affect BDEs’ size, thus indicating differential expression of molecular cargo.

### 3.2. RNA Seq and Bioinformatics Analysis

Having ascertained the isolation and characterization of BDEs, total RNA was isolated from P14 BDEs from the three groups of offspring, saline, PNO, and IUO, and subjected to miRNA sequencing using an RNA-Seq approach to identify differentially expressed BDEs miRNAs for IUO and PNO groups (Table 1).

**Figure 2:**
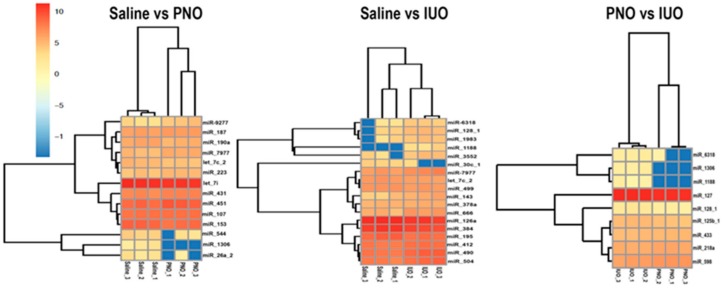
BDEs miRNAs signatures for IUO and PNO offspring. 2D hierarchical clustering heatmaps for the significant miRNAs and samples in the three comparisons, IUO vs. PNO, IUO vs. saline, and PNO vs. saline. The horizontal axis shows the clustering of samples within the two groups of each comparison, and the vertical axis represents the clustering of the significant miRNAs (*p* ≤ 0.05) for each comparison.

A 2D hierarchical clustering heatmap for the significant miRNAs (*p* ≤ 0.05) between the three groups is shown in Figure 2. Further employing a fold change of 1.5-fold up- or downregulated, we found eight miRNAs (four up- and four downregulated) in the PNO offspring. The IUO offspring had the highest number of significantly altered BDEs miRNAs (12), of which eight were upregulated and four downregulated compared to the controls. Further comparing the IUO to the PNO group, we found four BDEs miRNAs that were all upregulated in the IUO offspring.

**Figure 3:**
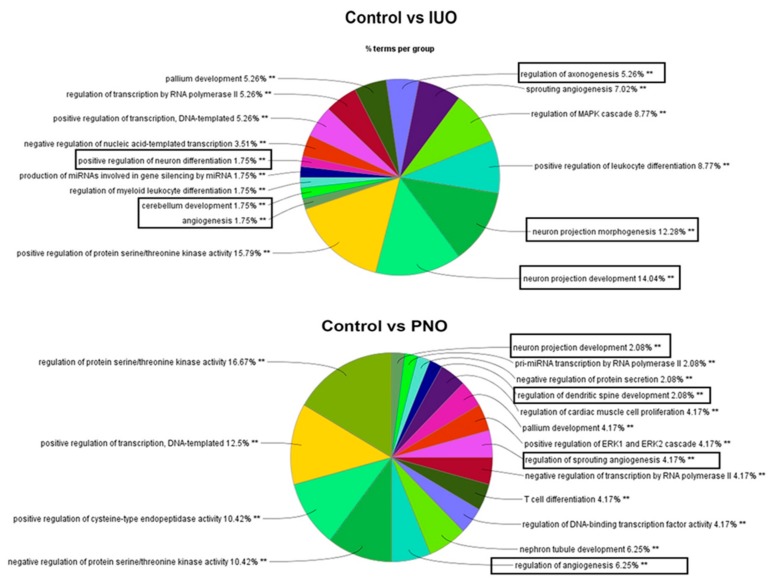
Mapping of biological processes. Clue-Go analysis of differentially expressed miRNAs targeted gene functions in the PNO and IUO groups. Pie diagrams show enriched biological processes involved in developmental, neurological, and psychological disorders (boxed), which are more impacted in the IUO offspring. The asterisks represent the group term *p*-value representing each category. ** *p* < 0.001.

Next, using a bioinformatics approach, we mapped the differentially expressed miRNAs to identify their target genes, including the enriched cellular processes. Since the experimentally known targets were limited, we also used high-confidence target predictions by TargetScan using ingenuity pathway analysis (IPA) (Supplementary Table S1) as well as the enriched cellular processes for the different treatment groups (Supplementary Table S2) using ClueGo. As seen in Figure 3 we found the highest enrichment of gene ontology (GO) terms involved in neurodevelopment such as neuron projection development, neuron projection morphogenesis, pallium development, cerebellum development, and positive regulation of neuron differentiation in the IUO offspring. While some GO terms involved in neuron projection development were also enriched in the PNO vs. saline and IUO vs. PNO comparisons, the enrichment was much higher in the IUO pups. Taken together, these data indicate that the IUO offspring demonstrate significant alterations in the BDEs cargo, including enhanced impairments in key brain development functions. 

### 3.3. BDEs from IUO Offspring Enhance Synaptodendritic Damage 

**Figure 4:**
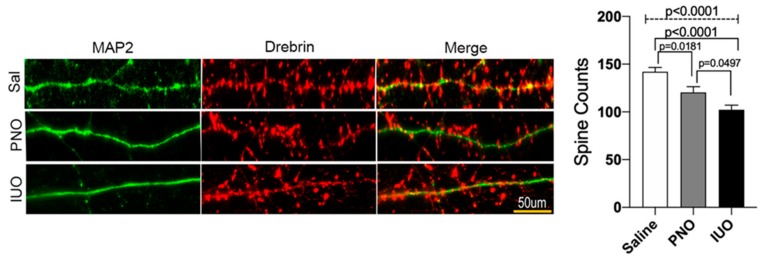
Confocal imaging of dendritic spines. Representative images of dendritic spines (50 µm) from saline, PNO, and IUO BDEs treated (for 24 h) DIV14 primary rat cortical neurons. Neurons were immunostained for MAP2 (neuronal marker, green) and Drebrin (dendritic spine marker, red). Histograms show the overall spine counts for the three groups. Confocal images were processed by ImageJ and the number of dendritic spines on 50-µm-long dendrites were manually counted. All the experiments were performed in three replicates (*n* = 3) and a total of 10 neurons from each replicate were used for analysis. For the final compilation of the data, 50 μm dendrite segments and 45 dendrites per condition were used for the final analysis. Error bars = SEM. One-way ANOVA (hatched line) with Tukey’s multiple comparison test was used to determine significance. *p*-values are represented above for each comparison.

Following up on our bioinformatics outcome on alterations in key brain functions during development in IUO offspring, we hypothesized that these BDEs from the offspring would also induce alterations at the synapse. To determine if BDEs impacted the synaptodendritic architecture, we treated 14 DIV rat cortical primary neurons with equal amounts of P14 BDEs from the three groups. Confocal imaging of dendritic spines using the neuronal and spine markers, MAP2 and Drebrin, respectively, showed a significant reduction upon treatment with PNO BDEs, which was further exacerbated in the IUO BDEs treatment group (Figure 4). Thus, these data denote that P14 BDEs from PNO and IUO offspring carry cargo that subsequently damages the synaptodendritic architecture and could further lead to neuronal dysfunction at a key stage of neurodevelopment. 

### 3.4. DRD1 Receptor Is Upregulated in Synaptosomal Fractions in the IUO Group

**Figure 5:**
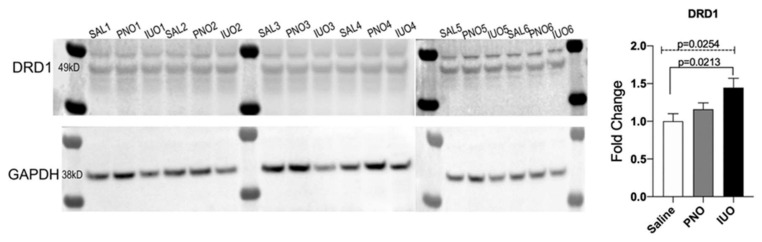
Validation of DRD1 expression, Western blot analysis of crude synaptosomal extracts isolated from saline (SAL), PNO, and IUO groups for dopamine receptors DRD1. GAPDH was used as an internal control. One-way ANOVA (hatched line) with Tukey’s multiple comparison test was used to determine significance. *p*-values are represented above for each comparison.

Because synaptodendritic injury was exacerbated in the IUO group, we wondered whether any specific miRNAs lead to such an effect. Through bioinformatic analysis, we found one miRNA, miR-504, to be upregulated in the IUO group. Previously, it has been shown that miR-504 can bind to the DRD1 receptor directly and modulates its expression and indirectly affects DRD2 expression [22]. Crude synaptosomal extracts were isolated from the P14 cortices and western blotting was conducted. Results indicated a significant increase in DRD1 expression in the IUO group when compared to saline controls (Figure 5). 

## 4. Discussion

Our current study using a preclinical model has identified novel BDE-derived miRNA signatures for altered neurodevelopment in PNO and IUO offspring. Opiates can pass into the placenta and act on fetal opioid receptors [23,24,25]. While our PNO group was exposed to oxy only via the breastmilk from birth until the weaning date, the IUO group was exposed via placental concentrations of oxycodone throughout gestation as well as via the breastmilk postnatally until weaning. Additionally, these groups are clinically relevant. Oxycodone is an opioid analgesic that can be prescribed during and after pregnancy. Oxycodone, marketed as OxyContin, is also one of many commonly misused or abused opioids. Because the IUO dam’s treatment starts before breeding, the IUO group represents women who exhibit chronic opioid use before, during, and after pregnancy. The paradigm of prenatal opioid exposure, whether the opioid is morphine, buprenorphine, or oxycodone, etc., is well established in the literature. Oxycodone as a postoperative analgesic has been reported in the literature for postpartum pain or caesarian sections in lieu of morphine drips [26,27]. The PNO group is representative of the human scenario in which a mother undergoes a caesarean section and is prescribed oxycodone for postpartum pain. While prenatal exposure paradigms are well represented in the field, postnatal exposure has not been as thoroughly investigated as of yet. 

The model system and the dose of oxy (15 mg/kg) used in our study mimic a chronic prescription experienced by opiate-dependent woman during gestation and parturition. This dose has been shown to be well tolerated in animals, induce behavioral deficits in the exposed offspring [12], and mimic the development of chronic analgesia in human subjects experiencing breakthrough pain (FDA PI. Oxycodone. https://www.drugs.com/pro/oxycodone.html Accessed 1 August 2017; Oxycodone. https://www.drugs.com/monograph/oxycodone.html Accessed 1 August 2017). Additionally, because the 15 mg/kg dose given to the dams is above what is generally prescribed to women post-caesarean section, the PNO group is also representative of neonates in neonatal intensive care units. Neonates with congenital abnormalities are exposed to high-dose opiate infusions post-delivery while in critical care units [28]. Infants with heart defects and persistent pulmonary hypertension are exposed to potent opiates for sedation and analgesia while supported by extracorporeal membrane oxygenation (ECMO) and mechanical ventilation [29]. In some cases, infants are supported by ECMO for three weeks or longer [30]. Also important to note is that the use of opiates in the NICU has been associated with increased diagnoses of neonatal abstinence syndrome [28]. A gap in knowledge exists regarding the implications of these opiate drips and how they may impact neurodevelopment in newborns. Our PNO group is clinically relevant as it provides a high-dose opiate exposure to the pups that is comparable to the high-dose opiate exposure neonates in the NICU experience.

The degree of exposure of an infant to a drug passed through the breastmilk depends on the concentration of the drug in the milk, the amount of milk ingested, and the rate of elimination from the infant [31]. Not measuring the concentration of oxy in the breastmilk and plasma of our dams or determining the concentration of oxy in the plasma of our IUO and PNO pups is a limitation in our study. However, a human study by Seaton et al. has shown that oxy is concentrated in the breastmilk and offspring exposed via the breastmilk may receive less than 10% of a typical therapeutic oral infant dose (0.1–0.2 mg/kg) [32,33,34,35,36]. Despite this low dose, infant exposure to oxy via the breastmilk has been associated with central nervous system depression [37], and a number of animal studies have also revealed deficits in behavior and development associated with perinatal opioid exposure [12,38,39,40]. Additionally, opiates can pass into the placenta and act on fetal opioid receptors [23,24,25]. While our PNO group was exposed to oxy only via the breastmilk from birth until the weaning date, the IUO group was exposed via placental concentrations of oxycodone throughout gestation as well as via the breastmilk postnatally until weaning.

Recent studies coupled to high-throughput technologies have further provided new insights into the molecular underpinnings associated with chronic oxy dependency. These include alterations in genes associated with the integrated stress response in the brain [41], induction of apoptotic signaling in neurons by promoting demyelination [39], alterations in reward-related genes [42], axon guidance molecules [42], inflammation/immune-related genes [43], neurotransmitter receptor genes [44] as well as expression of synaptic plasticity genes [45], including key sex-specific neuroplasticity-related genes [46]. miRNAs represent a class of noncoding RNAs that critically regulate gene expression by binding to the 3′ UTR of target transcripts, thus blocking their translation. Several studies have documented the role of these miRNAs in a plethora of diseases, including maladaptive behavior associated with addiction to drugs of abuse [47,48,49]. In recent years, several important studies have found that EVs, which express a repertoire of cargo, including miRNAs [10], can function within the recipient cell to alter protein expression [9,50], triggering inflammatory responses and neuronal damage [8,51].

However, there is a paucity of studies on how chronic oxy dependency during and after pregnancy can impact BDEs miRNAs expression and subsequently induce synaptic deficits in the exposed offspring. Accordingly, we performed RNA-Seq analysis on BDEs from postnatal day 14 (P14) brains, a key stage of brain development associated with synaptogenesis, from the three different groups. Interestingly, we found a significant difference in BDE sizes as evident by TEM (Figure 1B). An altered size distribution could highlight the enrichment of specific EV subpopulations. Results indicated that BDEs isolated from the saline group consisted of a majority of small EVs (~50–100 nm) whereas BDEs isolated from both IUO and PNO groups revealed larger EVs (>200 nm). It is well known that drugs and comorbidities can influence the mechanisms of biogenesis and release of EVs [52]. Here, we speculate that oxy exposure perturbed the EV biogenesis pathway and therefore EV sizes. Further, RNA sequencing was performed on the BDEs. The IUO offspring showed the greatest number of altered BDE miRNAs compared to the controls. Among these, miRs 6318, 666, 128-1-5p, 143-5p, 3552, 490-3p, 1983, 1188-3p, and 504 were upregulated and miRs 384-5p, 30c-1, and 195-5p were downregulated. While the roles for miRs 6318 and 666 are currently very limited, upregulation of miR-128-1-5p in the prefrontal cortex in mice exposed to chronic mild stress has been implicated in the etiology of depression [53]. A similar increase of this miRNA in our IUO offspring implicates that these offspring are potentially more vulnerable to depression at later stages of development.

On similar lines, we also identified miR-504 to be upregulated in the IUO offspring. In the human genome, miR-504 is an intronic miRNA, hosted by the fibroblast growth factor 13 gene (*FGF13*) on chromosome Xq26.3, which is highly expressed in the brain and was previously associated with Börjeson–Forssman–Lehmann syndrome, an X-linked mental retardation [54]. MiR-504 is expressed in cortical and hippocampal regions of the human brain, regulates the density of dendritic spines in the cultured hippocampal neurons [55], and is upregulated in postmortem brains of bipolar disorder patients [55] as well as in a rat model of cocaine-induced conditioned place preference [56]. Another study demonstrated that maternal deprivation enhances behavioral vulnerability to stress during adulthood, which is associated with the upregulation of miR-504 and downregulation of dopamine receptor D2 (DRD2) expression in the nucleus accumbens [22]. Similarly, a recent clinical study provides new evidence on the presence of a functional polymorphism in the dopamine receptor D1 (DRD1) to be modulated by miR-504 and associated with depression [57]. Furthermore, miR-504 expression has been shown to be significantly associated with depressive behaviors in stressed rats [22].

In our study, we observed an increase in miR-504 levels in BDEs isolated from the IUO group. Since miR-504 has also been shown to increase the DRD1 receptor [58], we looked at the expression of the DRD1 receptor in the synaptosomal fractions extracted from the cortices of the different treatment groups. Western blot analysis revealed an upregulation in the expression of the DRD1 receptor in both IUO and PNO offspring compared to the control but was significant only in the IUO. While in most of the literature an increase in miRNA expression is associated with downregulation of the gene target, this is not always the same. Since protein translation can be specifically targeted, the mRNA level can be unaffected and thus plausibly supports our current observation. DRD1 receptors are highly concentrated in dendritic spines, including spine heads, and the postsynaptic density of neurons [59], where they can interact with other receptors and influence the signaling mechanisms involved in the function of spines [60]. As noted above, since miR-504 has been associated with depression, a positive correlation of upregulation in D1DR expression could possibly serve as a compensatory effect in tuning cortical networks associated with behavior. Future studies using genetic approaches could potentially help our understanding of the downstream mechanisms associated with such a type of regulation.

Among the downregulated BDEs miRNAs in the IUO offspring, which included miRs-30c-1 (–0.17 fold), 384-5p (–1.56 fold), and 195-5p (–1.54 fold), miR-30c-1 downregulation is associated with a decrease in adult neurogenesis [61,62] as well as temporal lobe epilepsy in the latent phase in the rat pilocarpine model [63]. Downregulation of miR-384-5p has been shown to affect long-term synaptic and spine plasticity [64]. In another study, Ignacio et al., in a model of fetal alcohol spectrum disorders, showed that social enrichment with alcohol-naïve rats reversed the downregulation of miR-384-5p expression in animals prenatally exposed to ethanol [65]. Since drugs of abuse, including alcohol, cause alterations in neuroplasticity [66], it can be inferred that downregulation of miR-384-5p in the IUO offspring possibly indicates alterations in the spine and synaptic architecture, including behavioral deficits. Lastly, miR-195-5p downregulation has been shown to dysregulate the function of the chemokine CCL4 associated with inflammation in the etiology of focal cortical dysplasia [67]. This downregulation in the IUO offspring points to alterations in the inflammatory cascade in these offspring, which can subsequently impact brain function.

Another interesting finding in our study is the elucidation of the changes in BDEs miRNAs in the PNO offspring. Emerging evidence shows that new mothers tend to have a high prevalence of illicit drug use associated with postpartum depression and postoperative pain [68]. Interestingly, we identified some new signatures of BDEs miRs- 9277, 7977, and 451-5p to be upregulated while miRs- 544-3p, 26a-2, let7c-2, 190a-5p, and 1306-5p were downregulated in the PNO offspring. Among these, miR-7977 is upregulated in the EVs isolated from mesenchymal stem cells and linked to aberrant hematopoiesis in myeloid neoplasma [69]. Its upregulation in the BDEs of PNO offspring could potentially implicate its role in dysregulating neurogenesis during a critical stage of brain development in these offspring. Furthermore, environmental stressors, such as drugs of abuse, can impact mitochondrial dynamics during neurogenesis, which eventually can lead to impaired neurodevelopment due to altered energy metabolism. One in vitro study demonstrated that high glucose levels caused a significant increase in miR-451 levels, which led to enhanced proliferation of glioma cells, while an opposite effect was observed with low glucose levels [70]. In our study, an increase in miR-451 levels in PNO offspring could indicate altered energy metabolism associated with impaired mitochondrial dynamics in these offspring.

Among the downregulated hits, we found a profound decrease (–11 fold) in the expression of miR-26a-2, which has been extensively studied in gliomas. From a neurodevelopmental perspective, downregulation of miR-26a-2 has been linked to impaired long-term potentiation and spine dynamics [64] as well as the proliferation of pediatric brain tumors [71]. Another study showed its downregulation in the hippocampus of peripubertal rats exposed to binge ethanol exposure [72], suggesting that these PNO offspring are possibly prone to alcohol addiction later in development. Another important hit identified in the PNO offspring is let-7c-2, which regulates many cellular functions, including neuronal differentiation, cell subtype specification [73], neuronal regeneration [55,74], and synapse formation [75,76]. Downregulation of let-7c has also been associated with increased inflammation in the etiopathology of traumatic brain injury whereas treatment with let-7c mimics has been shown to attenuate inflammation [77]. Currently, there is no information on whether PNO offspring have a neuroinflammatory state in their brains. Based on the current literature and our own in vitro data showing spine loss after treatment with BDEs, it could be hypothesized that downregulation of let-7c in the BDEs could possibly lead to increased inflammation, resulting in neuronal impairment and synapse dysfunction in the PNO offspring during synaptogenesis. On these lines, miR-190a-5p downregulation in the spinal cord has been associated with diabetic neuropathic pain in a rat model [78]. The authors provide evidence that miR-190a-5p downregulation causes an increase in the expression of its target, vesicular glutamate transporter 2 (vGLUT2), and induces the expression of the inflammatory cytokines IL-6 and IL-1β. vGLUT2 is important for packaging the excitatory neurotransmitter glutamate into synaptic vesicles and plays a key role during critical periods of neurodevelopmental plasticity [79,80,81,82]. More recently, one study showed that downregulation of a cluster of miRNAs, which included miR-190a, is associated with postoperative cognitive dysfunction in response to anesthetics, such as isoflurane and sevoflurane, via the GABAergic transmission pathway [83]. These above observations lend distinct support to our current findings that miR-190a downregulation in PNO offspring could potentially lead to alterations in both excitatory and inhibitory neurotransmission pathways.

Although our study did identify a unique set of BDE miRNAs signatures associated with IUO and PNO exposure, we identified four hits that overlapped between our two treatment groups. All the four miRs, –6318, 1188, 1306-5p, and 128-1-5p, were upregulated in the IUO group compared to the PNO. While there is no existing scientific literature on miR-6318 to date, miR-1188 has been implicated in the etiology of memory disorders in a rat model of temporal lobe epilepsy, albeit its expression is downregulated in the hippocampus [84]. One possible explanation for this differential expression is the specificity of the brain region examined, the hippocampus, versus our study, which used the entire brain. Lastly, upregulation of miR-1306 levels has been shown to inhibit the expression of the Alzheimer-related gene *ADAM10* [85] while an increase in miR-128-1-5p levels has been associated in the etiopathogenesis of depression [53] as described in our earlier part of the discussion.

## 5. Conclusions

To summarize, this current study identified unique as a well as overlapping BDE miRNAs signatures at a key stage of brain development in IUO and PNO offspring. We further identified that the miR-504 increase in BDEs from the IUO group correlated with increased expression of DRD1 and DRD2 receptors in synapses, possibly a mechanism underlying the loss of dendritic spines. Further validation of various BDEs miRNAs signatures and associated key gene targets regulating synaptic function and subsequent neurodevelopment are well poised for future experimental studies.

## Figures and Tables

**Table 1:** List of differentially expressed miRNAs among the Control, PNO and IUO groups.

**PNO vs. Control**				
**miRNA**	**Seed sequence**	**up/down**	**Fold Change (PNO/Control)**	***p*-value (student’s *T*-test)**
rno-miR-1306-5p	CCACCTCCCCTGCAAACGTCCA	down	-inf	0.018
mmu-miR-26a-2-3p_1ss4GA	CCTATTCTTGATTACTTGTTTC	down	0.09	0.021
rno-miR-544-3p_R+1	ATTCTGCATTTTTAGCAAGCTT	down	0.29	0.018
rno-let-7c-2-3p	CTATACAATCTACTGTCTTTCC	down	0.64	0.015
rno-miR-190a-5p_R+1	TGATATGTTTGATATATTAGGTT	down	0.66	0.048
rno-miR-451-5p	AAACCGTTACCATTACTGAGTT	up	1.59	0.035
hsa-miR-7977_1ss6AG	TTCCCGGCCAACGCACCA	up	1.88	0.006
efu-mir-9277-p3_1ss9AT	CAGGTTCGTATCCTGCCGACT	up	2.64	0.023
**IUO vs. Control**				
**miRNA**	**Seed sequence**	**up/down**	**Fold Change (IUO/Control)**	***p*-value (student’s *T*-test)**
rno-miR-30c-1-3p	CTGGGAGAGGGTTGTTTACTCC	down	0.23	0.039
rno-miR-384-5p	TGTAAACAATTCCTAGGCAATGT	down	0.63	0.013
rno-miR-195-5p_R+1	TAGCAGCACAGAAATATTGGCA	down	0.64	0.047
rno-miR-504_L-1R+1	GACCCTGGTCTGCACTCTGTCT	up	1.84	0.039
rno-miR-490-3p_R+1	CAACCTGGAGGACTCCATGCTGT	up	1.93	0.016
hsa-miR-7977_1ss6AG	TTCCCGGCCAACGCACCA	up	1.98	0.044
rno-miR-666-5p_R+4	AGCGGGCACGGCTGTGAGAGCCCC	up	2.23	0.014
rno-miR-143-5p_R+1	GGTGCAGTGCTGCATCTCTGGT	up	2.76	0.024
rno-miR-128-1-5p_R+2	CGGGGCCGTAGCACTGTCTGAGA	up	3.19	0.019
mmu-mir-1983-p5_1ss1GA	AAAAGCATGCTCCAGTGGCGC	up	3.23	0.037
rno-miR-3552	AGGCTGCAGGCCCACTTCCCT	up	3.32	0.037
rno-miR-6318_R+1	CTGCCTGGCGCAGGGCCTGTAGT	up	4.60	0.003
rno-miR-1188-3p_L+2R+5	TCCGAGGCTCCCCACCACACCCTG	up	inf	0.012
**IUO vs. PNO**				
**miRNA**	**Seed sequence**	**up/down**	**Fold Change (IUO/PNO)**	***p*-value (student’s *T*-test)**
rno-miR-128-1-5p_R+2	CGGGGCCGTAGCACTGTCTGAGA	up	2.16	0.005
rno-miR-6318_R+1	CTGCCTGGCGCAGGGCCTGTAGT	up	6.29	0.007
rno-miR-1306-5p	CCACCTCCCCTGCAAACGTCCA	up	inf	0.001
rno-miR-1188-3p_L+2R+5	TCCGAGGCTCCCCACCACACCCTG	up	inf	0.012

Note: the cutoff criteria of *p*-value ≤ 0.05 and fold change of 1.5-fold up/down were used.

## Supplementary Materials

The following are available online at https://www.mdpi.com/2073-4409/9/1/21/s1, Table S1: Target Scan analysis using ingenuity pathway analysis was used to identify the gene targets for the differentially expressed BDE miRNAs. Table S2: Clue-Go gene function mapping to identify the various enriched cellular processes.
